# MOTS-C levels ın ındividuals with and without obesity and ıts association with ınflammation, insulin resistance and endothelial dysfunction

**DOI:** 10.20945/2359-4292-2025-0063

**Published:** 2025-09-22

**Authors:** Duygu Yildiz Ozkaya, Cem Haymana, Ibrahim Demirci, Umut Göktan Duman, Erhan Küpçük, Gizem Esra Koç, Ilker Tasci, Yusuf Alper Sonmez

**Affiliations:** 1 Department of Internal Medicine Gulhane, Faculty of Medicine, University of Health Sciences, Ankara, Türkiye; 2 Department of Endocrinology and Metabolism, Guven Hospital, Ankara, Türkiye

**Keywords:** Obesity, Insulin resistance, Mots-C Peptide

## Abstract

**Objective:**

To investigate the Mitochondrial Open Reading Frame of the 12S rRNA type-c (MOTS-c) peptide levels in individuals with obesity compared to those with a normal body mass index and to examine the association of MOTS-c levels with markers of insulin resistance, endothelial function, and inflammation.

**Methods:**

In this study 85 individuals were enrolled, including 48 with a body mass index ≥ 30 kg/m^2^ and 37 with a body mass index between 18.5 and 24.9 kg/m^2^. Individuals with smoking, pregnancy, type 2 diabetes mellitus and other chronic conditions were excluded. Blood samples were collected after at least 8 hours of fasting to measure serum MOTS-c, insulin, high-sensitivity C-reactive protein, and asymmetric dimethylarginine levels. Statistical analyses included t-tests, Mann-Whitney U tests, Chi-squared tests, correlation analyses, and multiple regression analyses.

**Results:**

We found no significant difference in serum MOTS-c levels between individuals with obesity and those with normal body mass index (14.33 ± 3.76 pg/mL *versus* 13.67 ± 3.44 pg/mL; p = 0.395). Serum MOTS-c levels showed a significant positive correlation with the HOMA-IR index (p < 0.05) but did not correlate with high-sensitivity C-reactive protein or asymmetric dimethylarginine levels. Multiple regression analysis indicated that age and HOMA-IR were significant predictors of MOTS-c levels, with MOTS-c decreasing with age and increasing with higher insulin resistance.

**Conclusion:**

Serum MOTS-c levels were similar in individuals with obesity and those with normal weight. The study highlighted age and insulin resistance as significant determinants of MOTS-c levels.

## INTRODUCTION

Obesity is a significant health issue worldwide. Genetic and environmental factors underlying obesity are often investigated, and new treatment options are often explored. As such research has shown, various factors such as hormones, genes, environmental chemicals, and socioeconomic status play a role in the development of obesity (^1^).

At the cellular level, various mechanisms also contribute to obesity’s development (^2^,^3^). Mitochondria - the organelles responsible for cellular energy production - possess a semi-autonomous genetic system and an independent genome (^4^). Mitochondrial DNA (mtDNA), which resembles bacterial circular DNA, encodes 37 genes, including 22 transfer ribonucleic acids (tRNA), two ribosomal ribonucleic acids (rRNA), and 13 messenger ribonucleic acids (mRNA) (^5^). While the mitochondrial genome encodes proteins within the organelle, proteins encoded by the nuclear genome are also imported to mitochondria. Mitochondrial Open Reading Frame of the 12S rRNA type-c (MOTS-c) is a mitochondrial peptide derived from a small open reading frame found in the 12S rRNA region in humans (^6^). Consisting of approximately 16 amino acids, MOTS-c has been reported to be involved in the regulation of cellular metabolism (^6^).

Given that mitochondria are the most important metabolic organelles in the body, it is unsurprising that MOTS-c exhibits global metabolic effects. It is found in the circulation (^7^) and has been shown to be active in regulating skeletal muscle functions (^8^) and glucose metabolism (^9^). Beyond that, MOTS-c is implicated in the regulation of obesity, diabetes, exercise, and longevity and represents a completely new mitochondrial signaling mechanism that regulates interand intracellular metabolism (^10^).

Despite data amassed in preclinical studies on mechanisms of obesity (^1^-^3^), whether the blood level of MOTS-c relates to body weight and obesity-related circulating biomarkers remains largely unknown. Therefore, in our study, we aimed to investigate the MOTS-c peptide levels in individuals with obesity compared to those with a normal body mass index (BMI) and to examine the association of MOTS-c levels with markers of insulin resistance, endothelial function, and inflammation.

## MATERIALS AND METHODS

### Study design

This cross-sectional, case-control study with prospective enrollment was carried out between the dates of January 1, 2022, and May 5, 2022, in a tertiary care hospital internal medicine outpatient clinic. The institutional review board of Gulhane Training and Research Hospital approved the study protocol. All procedures followed adhered to the Declaration of Helsinki, and all participants provided their signed Informed Consent.

### Study population

The study included patients with BMIs ≥30 kg/m^2^ in the study group and patients with BMIs of 18.5 to 24.9 kg/m^2^ in the control group. The exclusion criteria were smoking, type 2 diabetes mellitus, hypertension, coronary heart disease, stroke, peripheral arterial disease, acute or chronic kidney disease, rheumatic disease, asthma, cancer, or pregnancy. Patients with a history of acute conditions (i.e., infections, trauma, or surgery) were also excluded from the study.

### Anthropometric variables and blood pressure measurement

Height and weight were recorded with the patients in their underwear according to the standard protocol. Body mass index was computed as the ratio of weight to height squared (kg/m^2^). Arterial blood pressure was recorded using automatic blood pressure monitors (Iron, AS-35K) with patients sitting after at least 5 minutes rest. Three consecutive readings were performed on the same arm, and the mean was recorded.

### Laboratory variables

Blood samples were collected from patients after a fasting period of at least 8 hours. Blood was centrifuged at 2,000 × *g* for 10 minutes within 2 hours of collection, and supernatant serum was collected. Serum samples were stored at -80 °C until analysis.

Serum insulin level was measured using ELISA (ELISA Kit for Insulin [INS], Product No. CEA448Ra, USCN, Wuhan, China; sensitivity: minimum detectable concentration ≤ 51.6 pg/mL, intra-assay coefficient of variation (IntraCV): < 10% and inter-assay coefficient of variation (InterCV): < 12%) to estimate insulin sensitivity during Homeostatic Model Assessment (HOMA) using the formula glucose (mg/dL) × insulin (mU/L)/405 (^11^).

Serum high-sensitivity C-reactive protein (hs-CRP) levels were measured with ELISA (High Sensitive ELISA Kit for CRP Reactive Protein [CRP], Product No. HEA821Hu, USCN, Wuhan, China; sensitivity: minimum detectable concentration ≤ 27.4 pg/mL, IntraCV <10% and InterCV <12%) as the marker of low-grade inflammation (^12^).

Serum asymmetric dimethylarginine (ADMA) level was measured using ELISA as well (ELISA Kit for Asymmetrical Dimethylarginine [ADMA], Product No. CEB301Ge, USCN, Wuhan, China; sensitivity: [minimum detectable concentration] ≤ 4.99 ng/mL, IntraCV < 10% and InterCV < 12%) as the marker of endothelial dysfunction (^13^).

Last, serum MOTS-c level was also measured using ELISA (ELISA Kit for Mitochondrial Open Reading Frame of the 12S rRNA-c [MOTS-c], Product No. CEX132Hu, USCN, Wuhan, China; sensitivity: minimum detectable concentration ≤ 0.99 ng/mL, IntraCV <10% and InterCV <12%).

### Primary and secondary outcomes

The primary outcome was the difference in MOTS-c le^-^vels between individuals with obesity and normal weight. The relationship between MOTS-c level and hsCRP, ADMA, and Homeostatic Model Assessment of Insulin Resistance (HOMA-IR) were the secondary outcomes.

### Statistical analysis

Statistical Package for the Social Sciences (SPSS), version 26, was used for analyses. The normality of the distribution of continuous variables was tested with the Shapiro-Wilk test, and results were recorded as mean ± standard deviation for continuous variables and as n (%) for categorical ones. The demographic and clinical variables were compared using Student’s *t* test or the Mann-Whitney U test for normally distributed or skewed variables, respectively. The Chi-squared test was used to compared ratio variables between the groups, while the relationships between MOTS-c and the studied variables were examined using Pearson or Spearman correlation analysis in the whole, normal BMI, and obesity groups. Multiple regression analysis was performed to investigate factors associated with a high MOTS-c level. Significance was set at p < 0.05.

## RESULTS

### Patients’ characteristics

The study included 85 individuals (74.1% female, mean = 32.8 ± 11.3 years). The number of individuals in the normal BMI group was 37 and in the obesity group was 48. There were nine (18.9%) patients with severe obesity (i.e., BMI ≥ 40). The mean age of individuals with obesity was significantly higher than ones with normal BMI. The percentages of individuals 18 to 25 years old, 25 to 44 years old, and 45 years old or older significantly differed between the groups. Among patients with normal BMI and obesity, the mean BMI was 21.8 ± 1.8 and 36.0 ± 5.4 kg/m^2^, respectively (**Table 1**).

**Table 1:** Demographic characteristics and blood pressure comparisons between subjects with normal weight and obesity

	Normal BMI (n=37)	Obesity (n = 48)	Total (n = 85)	p-value
Age, years	28.0 ± 9.2	36.5 ± 11.4	32.8 ± 11.3	< 0.001
18-24	56,8	18,8	35,3	0.001
25-44	35,1	54,2	45,9	
45 or older	8,1	27,1	18,8	
Sex, female, n (%)	28 (75.1)	35 (73.2)	63 (74.1)	0.808
BMI	21.8 ± 1.8	36.0 ± 5.4	29.85 ± 8.2	< 0.001
Systolic blood pressure, mmHg	116.8 ± 11.5	128.04 ± 18.5	123.1 ± 16.7	< 0.001
Diastolic blood pressure, mmHg	70.8 ± 11.6	78 ± 12.9	74.8 ± 12.9	< 0.05

Results expressed as mean ± standard deviation, % or n (%).

BMI: body mass index.

### Laboratory findings

As shown in **Table 2**, compared with the normal BMI group, patients with obesity had higher levels of fasting plasma glucose (78.8 ± 6.6 *versus* 87.8 ± 13.3; p < 0.001), HbA1c (5.2 ± 0.3 *versus* 5.7 ± 0.3), HOMA-IR (1.8 ± 0.8 *versus* 3.1 ± 1.4; p < 0.001), hs-CRP (0.68 ± 0.64 mg/L *versus* 3.31 ± 5.20 mg/L; p < 0.001), and plasma ADMA levels (615.12 ± 138.42 *versus* 709.75 ± 160.68; p = 0.008). Serum MOTS-c levels did not differ between the groups with normal BMI and obesity (13.67 ± 3.44 pg/mL *versus* 14.33 ± 3.76 pg/mL; p = 0.395).

**Table 2:** Laboratory findings in subjects with normal body weight and obesity

	Normal BMI (n = 37)	Obesity (n = 48)	Total (n = 85)	p-value
Fasting blood glucose, mg/dL	78.9 ± 7.0	88.5 ± 13.8	84.24 ± 12.23	< 0.001
HbA1c, %	5.2 ± 0.3	5.7 ± 0.3	5.6 ± 0.4	< 0.001
HOMA-IR	1.74 ± 0.74	3.07 ± 1.38	2.48 ± 1.31	< 0.001
hs-CRP, mg/L	0.56 (0.44)	3.63 (5.94)	1.58 ± 3.95	< 0.001^*^
ADMA, ng/mL	628.71 ± 130.30	717.80 ± 161.27	678.20 ± 153.91	0.008
MOTS-C, pg/mL	13.67 ± 3.44	14.33 ± 3.76	14.04 ± 3.61	0.395

* Mann-Whitney U test.

Results expressed as mean ± standard deviation or median (interval interquartile).

BMI: body mass index; HbA1c: glycated hemoglobin; HOMA-IR: Homeostatic Model Assessment Insulin Resistance; hs-CRP: high-sensitivity C-reactive protein; ADMA: asymmetric dimethylarginine; MOTS-c: Mitochondrial Open Reading Frame of the 12S rRNA type-c.

Subgroup analyses also revealed no significant difference in serum MOTS-c levels among females with normal BMI and obesity or males with normal BMI and obesity (**Table 3**). HOMA-IR and ADMA levels were not statistically significant among males with obesity versus normal weight. MOTS-C levels also did not differ between normalweight females and females with obesity.

**Table 3:** Laboratory comparisons between women and men with normal body mass index and obesity

	Women				Men		
	Normal BMI (n = 28)	Obesity (n = 35)	p-value		Normal BMI (n = 9)	Obesity (n = 13)	p-value
Fasting plasma glucose	78.16 ± 5.85	86.43 ± 13.98	0.002		81.57 ± 10.18	94.70 ± 12.00	0.030
HbA1c, %	5.2 ± 0.3	5.7 ± 0.3	< 0.001		5.2 ± 0.2	5.8 ± 0.3	< 0.001
HOMA-IR	1.75 ± 0.68	3.12 ± 1.28	< 0.001		1.71 ± 1.00	2.91 ± 1.71	0.056
hs-CRP, mg/L	0.43 (0.81)	3.63 (5.73)	< 0.001^*^		0.74 ± 0.42	5.43 ± 4.77	0.010
ADMA, ng/mL	630.68 ± 133.96	727.81 ± 165.52	0.011		621.65 ± 125.97	688.77 ± 151.97	0.258
MOTS-C, pg/mL	14.42 ± 3.37	14.36 ± 3.84	0.797		10.99 ± 2.21	14.25 ± 3.73	0.112

* Mann-Whitney U test.

Results expressed as mean ± standard deviation or median (interval interquartile).

BMI: body mass index; HbA1c: glycated hemoglobin; HOMA: Homeostatic Model Assessment Insulin Resistance; hs-CRP: high-sensitivity C-reactive protein; ADMA: asymmetric dimethylarginine.

### Correlations and multiple regression analyses

As shown in **Table 4**, serum MOTS-c levels showed significant positive correlations with HOMA-IR values across the entire sample and among patients with obesity. Age, blood pressure, fasting plasma glucose, HbA1c, hsCRP, ADMA, and obesity classification, however, did not show univariate associations with the MOTS-c level.

**Table 4:** Correlations between MOTS-C level and clinical and biochemical variables

Variables	MOTS-C level							
	Total			Normal BMI			Obesity	
	r	p-value		r	p-value		r	p-value
Age	-0.136	0.222		0.049	0.779		-0.223	0.132
Systolic blood pressure	-0.050	0.660		-0.045	0.795		-0.234	0.118
Diastolic blood pressure	0.080	0.480		0.175	0.315		-0.015	0.920
BMI	0.021	0.853		-0.201	0.246		-0.092	0.538
Fasting plasma glucose	0.108	0.332		0.039	0.823		0.130	0.385
HbA1c	0.051	0.652		0.179	0.311		-0.083	0.588
HOMA-IR	0.373	0.001		0.201	0.247		0.470	0.001
hs-CRP	0.005	0.968		0.034	0.847		-0.136	0.378
ADMA	0.035	0.756		0.077	0.667		-0.029	0.851

BMI: body mass index; HbA1c: glycated hemoglobin; HOMA-IR: Homeostatic Model Assessment Insulin Resistance; hs-CRP: high-sensitivity C-reactive protein; ADMA: asymmetric dimethylarginine.

A simple linear regression and multiple regression analyses were performed to explore the variables that predict MOTS-c level based on statistical associations at p < 0.250 (**Table 5**). A significant regression equation was found, F(3, 78) = 7.817, p < 0.001 (R2 = 0.202). Participants’ predicted MOTS-c level was equal to 12.598 to 0.078 (age) + 1.191 (HOMA), in which age was entered in years and HOMA-IR was entered as the index value. In other words, MOTS-C level dropped by 0.078 ng/mL for each additional year of age and increased by 1.191 ng/mL for each unit increase in the HOMA-IR index. Adding obesity or BMI as a covariate to the model did not alter the association of age and HOMA-IR index value with serum MOTS-c level.

**Table 5:** Simple and multiple linear regression analyses showing variables associated with MOTS-C level

Model	Unstandardized coefficients		Standardized coefficients	t	Sig.	95,0% confidence interval for B^*^	
	B	Standard error	Beta			Lower bound	Upper bound
Simple linear regression							
Age	-0.044	0.036	-0.136	-1.230	0.222	-0.116	0.027
Sex, women	1.901	0.906	0.228	2.097	0.039	0.097	3.704
Systolic blood pressure	-0.011	0.026	-0.050	-0.441	0.660	-0.062	0.040
Diastolic blood pressure	0.023	0.033	0.080	0.709	0.480	-0.042	0.089
BMI	0.009	0.050	0.021	0.187	0.853	-0.091	0.109
Fasting plasma glucose	0.034	0.035	0.108	0.975	0.332	-0.035	0.103
HbA1c	0.489	1.082	0.051	0.452	0.652	-1.665	2.643
HOMA-IR	1.067	0.297	0.373	3.597	0.001	0.477	1.658
hs-CRP	0.005	0.118	0.005	0.040	0.968	-0.231	0.241
ADMA	0.001	0.003	0.035	0.311	0.756	-0.004	0.006
Obesity	0.691	0.830	0.093	0.833	0.408	-0.961	2.344
Multiple regression							
Age	-0.078	0.033	-0.238	-2.322	0.023	-0.144	-0.011
Sex, women	1.583	0.830	0.190	1.907	0.060	-0.069	3.236
HOMA-IR	1.191	0.295	0.416	4.043	< 0.001	0.604	1.777

* This column shows the 95% confidence interval (CI) for the B coefficient (effect size). BMI: body mass index; HbA1c: glycated hemoglobin; HOMA: Homeostatic Model Assessment Insulin Resistance; hs-CRP: high-sensitivity C-reactive protein; ADMA: asymmetric dimethylarginine.

## DISCUSSION

Our study revealed no significant difference in MOTS-c levels between individuals with normal weight and individuals with obesity. However, a significant positive correlation was found between MOTS-c and HOMA-IR levels. According to the multivariate analyses, age and insulin resistance were significant determinants of MOTS-c levels.

MOTS-c, a mitochondrial peptide, is believed to be involved in metabolic homeostasis and therefore thought to be associated with metabolic diseases such as diabetes and obesity (^10^,^14^). Although numerous studies have been conducted in animals, human studies on MOTS-c have remained limited, and the relationship between obesity and MOTS-c levels has not been fully elucidated. In a past study, MOTS-c levels were found to be lower in children with obesity than ones with normal weight, while a subgroup analysis by gender revealed lower levels in both male and female children with obesity (^15^). Another study conducted among adolescents revealed lower MOTS-c levels in patients with obesity, along with an inverse relationship between MOTS-c levels and obesity-related markers (^16^). However, because that relationship was not observed in female children, it has been hypothesized that ovarian hormones may interfere with the expression of MOTS-c (^16^). In another study conducted with 20 adults, MOTS-c levels were similar in patients with obesity and normal-weight individuals (^17^). Taken together, those studies have produced inconsistent results regarding the relationship between MOTS-c levels and obesity. Our findings are similar to the results of the study in which no difference emerged between patients with obesity and normal-weight individuals in terms of MOTS-c levels (^17^). It seems that alterations in MOTS-c levels occur more within the pediatric than the adult population.

We also found no significant difference in circulating MOTS-c levels between adults with obesity and ones with normal weight. That finding contrasts past results from studies conducted in pediatric populations, in which significant differences were observed based on weight status (^15^). That discrepancy may be attributed to age-related variations in mitochondrial function and MOTS-c regulation. During childhood and adolescence, growth and development are accompanied by unique metabolic demands and potentially more dynamic mitochondrial stress responses, which might lead to differential MOTS-c secretion across patients grouped according to BMI. By contrast, adults may exhibit more stable mitochondrial activity, and the presence of compensatory and/or adaptive physiological mechanisms may attenuate such differences. Furthermore, factors such as age, hormonal status, and prior use of medication among adults may further modulate the expression of MOTS-c, thereby contributing to a convergence of levels across categories of body weight. Those findings highlight the importance of considering age and developmental stage when interpreting MOTS-c data and underscore the need for longitudinal studies to better understand the regulation of MOTS-c across the lifespan.

Meanwhile, studies conducted with rats have suggested that MOTS-c exerts a protective effect against cardiovascular dysfunction (^18^). Therefore, the relationships between MOTS-c and inflammation, endothelial dysfunction, and insulin resistance have been investigated in a few studies. A study with children with normal weight and obesity showed a significant negative correlation of MOTS-c levels with insulin resistance and vascular endothelial damage (^16^). In a study conducted among adults, a positive correlation was found between HOMA-IR and MOTS-c levels; however, when those levels were examined separately in individuals with normal weight and obesity, no relationship was observed among patients with obesity, whereas a positive relationship was observed among normal-weight individuals (^17^). In another study, researchers compared MOTS-c levels among healthy individuals and individuals with non-alcoholic fatty liver disease, an important risk factor of cardiovascular diseases (^19^). MOTS-c levels were significantly lower among patients with fatty liver disease and ones with high scores for fibrosis, while a significant negative correlation surfaced between MOTS-c levels and metabolic syndrome.

In our study, we also aimed to test the relationship between MOTS-c levels and insulin resistance, inflammation, and endothelial dysfunction. A significant positive correlation was observed between MOTS-c levels and HOMA-IR levels, a surrogate marker of insulin resistance; however, no correlation arose between MOTS-c levels and hsCRP levels, an indicator of inflammation, and ADMA, a surrogate marker of endothelial function. At the same time, MOTS-c levels tended to decrease with advancing age and to increase with insulin resistance. It has been demonstrated in some studies that MOTS-c levels are associated with aging and that young people have higher MOTS-c levels than older adults (^20^,^21^) Previous studies have also revealed that MOTS-c may improve insulin sensitivity and glucose utilization in skeletal muscle (^16^,^22^). Our results also suggest that MOTS-c levels increase in parallel with increased insulin resistance. Along similar lines, in an animal study, MOTS-c therapy was administered to mice with gestational diabetes mellitus, which resulted in a significant improvement in hyperglycemia, insulin sensitivity, and glucose tolerance (^23^). Even so, the potential involvement of MOTS-c in the pathogenesis of diabetes mellitus remains unknown.

Compared with past studies investigating MOTS-c levels (^15^-^17^,^19^), our work introduced a distinct perspective by focusing exclusively on healthy adults with either normal BMI or obesity but devoid of any diagnosed comorbidities. Whereas earlier investigations have examined pediatric populations (^15^,^16^), individuals with hepatic steatosis (^19^), or smaller adult samples (^17^), our study included a broader, better-controlled cohort of adults. Furthermore, by excluding participants with chronic disease or inflammatory conditions and smokers, we aimed to reduce the influence of potential confounders on circulating MOTS-c levels. That methodological approach enhanced the reliability of our results and underscores their significance in contributing to a clearer understanding of MOTS-c dynamics specifically in metabolically uncomplicated obesity. Therefore, we believe that our findings provide a novel contribution to the literature and may serve as a foundation for future studies exploring the role of MOTS-c in early metabolic dysregulation.

Our findings have several limitations. First, due to the study’s cross-sectional design, it was impossible to establish causal relationships between MOTS-c levels and other parameters. Moreover, the sample size was not large enough. Inconsistent reports in the literature may be due to the fact that MOTS-c is a relatively new molecule, and therefore, measurement methods have not yet been standardized. Moreover, differences in the studied populations may also contribute to heterogeneous results. Despite those factors, we consider our findings to be important evidence demonstrating MOTS-c levels in patients with obesity and some of the first to reveal the relationship between MOTS-c and factors involved in atherosclerosis. Moreover, they may provide guidance for more comprehensive and/or extensive studies in the future.

In conclusion, our study revealed that serum levels of MOTS-c were similar in patients with obesity and normal-weight healthy individuals. Patients’ age and insulin resistance come to the fore as parameters affecting MOTS-c levels. However, prospective studies with larger samples are needed to better define the role of MOTS-c in obesity and metabolic diseases.

## Data Availability

all data are available from the corresponding author

## Data availability:

datasets related to this article will be available upon request to the corresponding author.
